# Quinsy

**Published:** 1881-03

**Authors:** Walter M. James

**Affiliations:** Phila.


### QUINSY.

BY WALTER M. JAMES, M.D., PHILA.

Among the diseases most frequently mal-treated by the rational therapeutics of the old school of medicine is quinsy.

Stormed at with mercury, leeches, blisters and poultices, the inflammation steadily advances, until suppuration occurs in a period of from eight to ten days. Treated homœopathically there are few ailments which so clearly demonstrate the truth of Hahnemannian principles when these latter are exclusively applied.

Depending, as it does, upon a scrofulous taint of the constitution any prescription made for the local trouble must cover the whole scrofulous condition by a careful attention to the totality of the symptoms, if we would be successful. A remedy accurately selected according to Hahnemann's directions, and therefore according to the inflexible logic of the law will cure the trouble before abscess has commenced to form. This is a most brilliant result, and one very gratifying to the patient and his friends. Yet we can not always attain this success. Notwithstanding our best efforts we fail to discover the simillimum and the inflammation proceeds to suppuration. Even in such case our remedies may not have failed to make a valuable impression upon the system. This will be apparent in the greater comparative freedom from trivial complaints after such attack; or if the quinsy be of periodical recurrence each successive attack will be less severe. This, however, is a very difficult lesson to impress upon the patient. If we do not prevent suppuration the patient considers our treatment a "failure."

The writer has had many cases of quinsy and most of them, from the above point of view may be considered "failures." Yet the two or three following cases being so strikingly different are considered worth relating.

In the summer of '78 a gentleman, having been overheated, sat down in a draft of air to become cool. Perspiration was suddenly checked and an attack of quinsy followed. The only reliable indication that appeared for the remedy was *profuse perspiration* out of all proportion to the heat of the weather. This perspiration was quite *oily*. Upon these considerations I gave merc. v. C M (Fincke.) In twelve hours he was relieved, and in twenty-four hours entirely cured without suppuration.

In Jan. '79 Mrs. H. S., who was a frequent sufferer from quinsy, the attack lasting generally eight to ten days, was seized with inflammation of the right tonsil. I failed to select the right remedy and the tonsil suppurated. One month later the same lady was affected in a similar way in the left tonsil. Again I failed and abscess began to form. A little further questioning brought out the following symptoms: flushes of heat, frequent waking from sleep at night, weak, faint feeling at the stomach. These will be recognized at once as the characteristics of sulphur. I gave sulphur 2 C and in twenty-four hours she was cured without the abscess maturing.

In March 1879 Mrs. B., a sister of the preceding, had quinsy of the left side. On doubtful indications I gave at first lachesis; but without avail. I then found heat, restlessness, and thirst at night. This would indicate aconite. But there was not that peculiar mental symptom of acon., "irresistible restlessness, fear, and agonized tossing about." Hence aconite failed and the suppurative process progressed. To my surprise I found that the heat was a series of flushes. That she slept in short "cat-naps," and that she had weak, fainty feelings. Here were sulphur symptoms. They had been present all the time but had not observed them. I immediately changed to sulphur 2 C which cured in twenty-four hours; the suppurative process ceasing immediately without discharge.

On Sat. Dec. 27, 1879, Miss T. S., subject to quinsy, was seized with an attack. There being no reliable indication except that it commenced on the right side with some tendency to the left, I gave Lyc. 2 C but it had no effect. The next day but one the tongue was red and the papillæ elevated. The tonsils were much swollen and very red. She had a constant desire to swallow which was very painful. I gave merc. jodat. rub. 10 M and in a few hours the abscess burst. This I believe to be due to the action of the remedy as formerly this patient would suffer from the abscess for a week before it would discharge.

On Jan. 20, 1881, this same young lady sent for me to remove a particle of sand or dust from the eye. Examination failed to discover any foreign matter. The eye, however, was much inflamed and swollen. I told her she had "taken cold;" but she insisted upon the presence of sand. The next day my diagnosis was confirmed. She sent for me again and I found a well-developed quinsy.

The indications were:

Inflammation commencing in the *left* tonsil.

Involuntary *loosening of the collar* around the the neck.

Severe headache commencing in the evening and lasting all night. It was made worse *whenever she fell asleep.*

The pain *commenced at the neck* and extended all over the head.

*Stiffness of the neck.*

These symptoms, though rather vague, pointed more strongly to lachesis than to any other remedy. I accordingly gave lachesis 2 M (Jenichen.) The next day when I called the symptoms had nearly disappeared. The inflammation of the tonsil was hardly noticeable and the headache much improved.

It is almost unnecessary to say that there was no subsequent suppuration of the tonsil.

